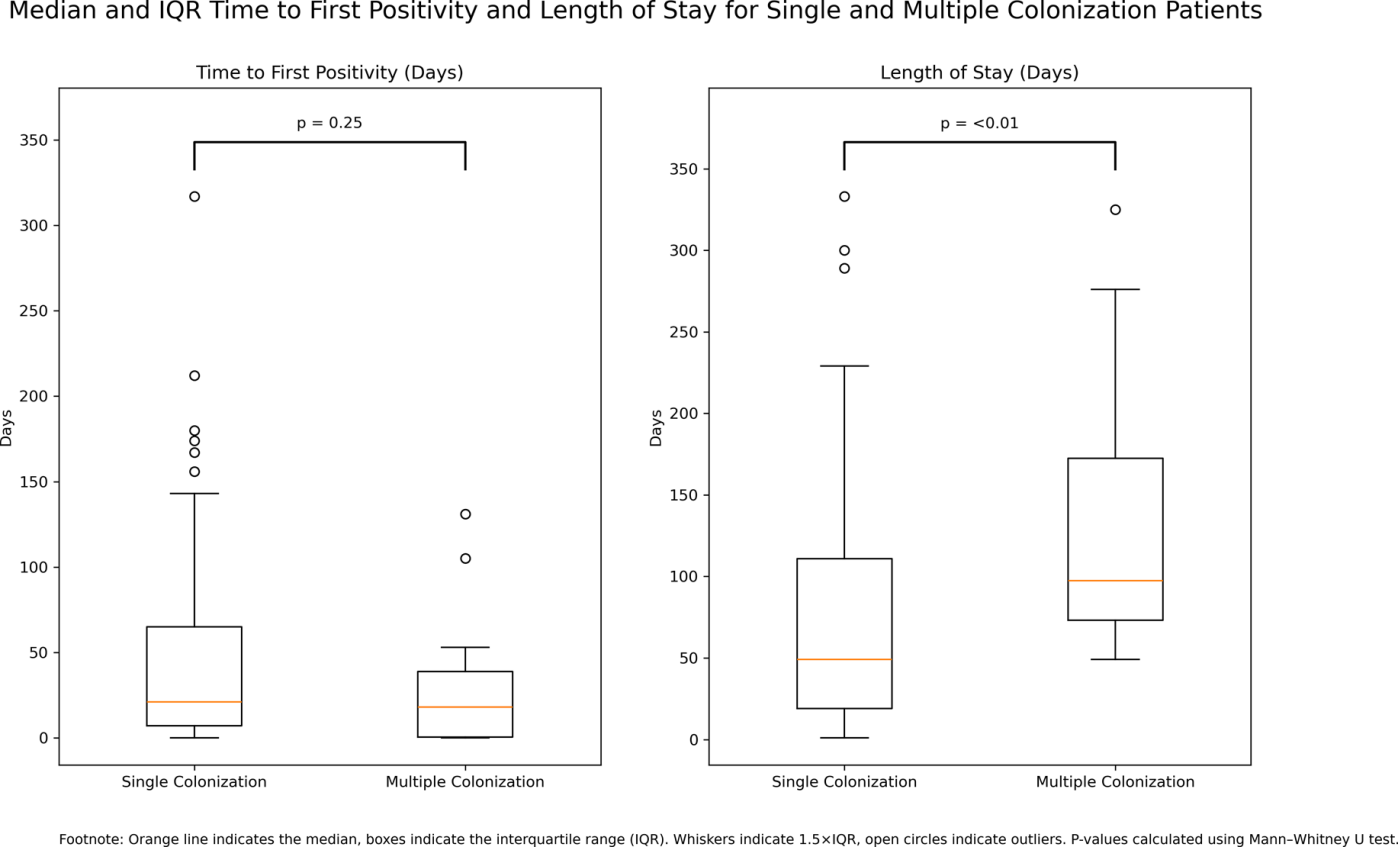# 347 Time and motion evaluation of disinfection practices before and after a switch from a dilutable to a ready-to-use disinfectant

**DOI:** 10.1017/ash.2026.10687

**Published:** 2026-06-23

**Authors:** Brian Alloway, Nicole McNeil, Jeannie Chan, Christy Pak, Jennifer Gantz, Carolyn Caughell, Nahid Hiermandi, Kim Stanley, Thomas Shimotake, Lynn Ramirez, Victoria Chu

**Affiliations:** 1 University of California, San Francisco; 2 University of California San Francisco; 3 Ucsf; 4 Benioff Children’s Hospital University of California San Francisco; 5 UCSF Health; 6 UCSF; 7 UC San Francisco

## Abstract

**Methicillin-resistant Staphylococcus aureus (MRSA) is associated with high morbidity, mortality, and outbreaks in neonatal intensive care units (NICU). For MRSA-colonized patients in the NICU, the Society for Healthcare Epidemiology in America recommends continuing contact isolation until NICU discharge, reflecting limited evidence to support safe discontinuation and recolonization concerns. We evaluated MRSA colonization dynamics in a NICU with an active surveillance program to better inform the evidence base for contact isolation practices. All patients in the 58-bed NICU at UCSF Benioff Children’s Hospital San Francisco undergo weekly or biweekly MRSA culture screening. Colonized patients are placed in single-patient rooms on contact isolation for the duration of their NICU admission and undergo decolonization with intranasal mupirocin and topical mupirocin or chlorhexidine wipes; parents are also offered decolonization. We conducted a retrospective review of MRSA surveillance data from January 1, 2021 through December 31, 2024. A patient colonization episode was defined as one or more consecutive positive screens, with multiple episodes separated by at least one negative screen. Comparisons between patients with single and multiple colonization episodes were performed using the Mann-Whitney U test. Among 2,352 patients screened, 111 (5%) had at least one positive MRSA screen and of these, 80 (72%) had a single positive screen. The median time to first positivity (TFP) was 20 days (interquartile range [IQR]:** 6-59) and the median length of stay (LOS) was 65 days (IQR: 24-134). Ninety-three (84%) patients had a single colonization episode and 18 (16%) had multiple colonization episodes (Figure 1). Compared with patients with one colonization episode, patients with multiple colonizations had similar median TFP (18 vs. 21 days; p=0.25), but a longer median LOS (98 vs. 49 days; p<0.01) (Figure 2). Colonized patients were placed on a cumulative 5,180 contact isolation days (median: 26 days per patient, IQR: 12-63 days); of these, 2,543 contact isolation days (49%) were during weeks with a negative screen. In a NICU with active MRSA surveillance, targeted decolonization, and robust horizontal infection prevention practices (e.g. hand hygiene, personal protective equipment, and environmental cleaning), MRSA colonization and recolonization detection was lower than previously reported in the literature. Despite this, contact isolation days accumulated substantially, with almost half of the days occurring during periods of negative surveillance testing. These findings highlight the need for further studies to assess MRSA recolonization risk and optimize the duration of contact isolation in NICU patients, ideally guided by local epidemiology and active surveillance.

**Figure:**
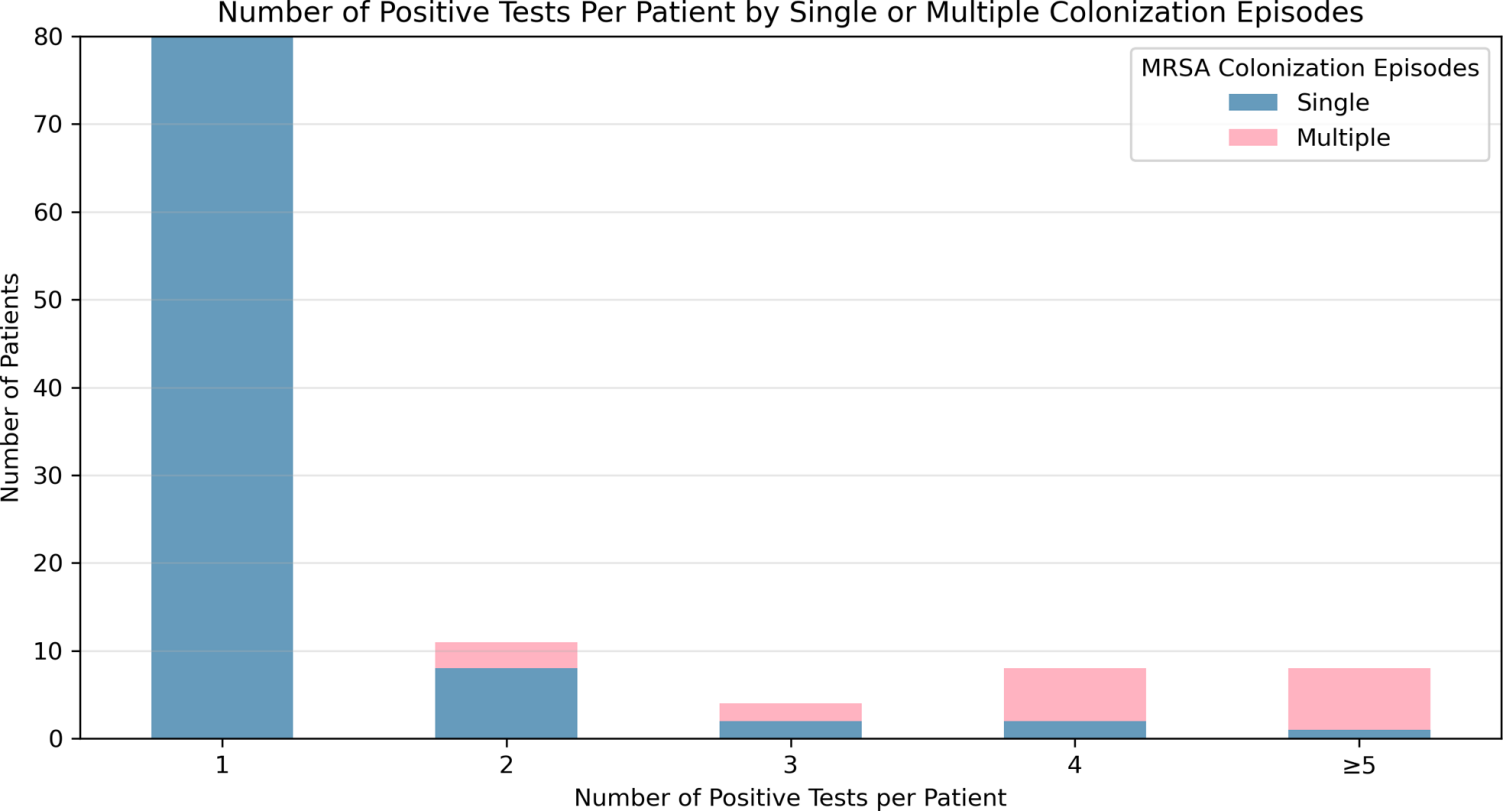

**Figure:**